# Laparoscopic intersphincteric resection versus an open approach for low rectal cancer: a meta-analysis

**DOI:** 10.1186/s12957-017-1304-3

**Published:** 2017-12-28

**Authors:** Hanyu Chen, Bin Ma, Peng Gao, Hongchi Wang, Yongxi Song, Linhao Tong, Peiwen Li, Zhenning Wang

**Affiliations:** Department of Surgical Oncology and General Surgery, The First Hospital of China Medical University, Shenyang, 110001 People’s Republic of China

**Keywords:** Intersphincteric resection, Rectal cancer, Laparoscopic, Meta-analysis

## Abstract

**Aim:**

The aim of this study was to compare the short-term and mid-term effects of laparoscopic intersphincteric resection with the conventional open approach for patients with low rectal cancer through a meta-analysis.

**Methods:**

The PubMed, EMBASE, Cochrane, and Ovid databases were searched for eligible studies until March 2017. Operation time, blood loss, circumferential resection margin-positive rate, distal margin length, number of resected lymph nodes, diverting stoma rate, postoperative overall morbidity, anastomotic leakage, and hospital stay were the main short-term effect endpoints. We also examined disease-free survival, overall survival, local recurrence, and post-operational anal function as secondary outcomes to evaluate the mid-term effects of laparoscopic surgery.

**Results:**

Five studies involving 620 patients were included in the analyses. Compared with the open approach, the laparoscopic ISR had less blood loss (weighted mean difference [WMD] = − 214.65 ml, 95% CI [− 370.44, − 196.13], *p* < 0.01), less postoperative overall morbidity (OR = 0.58, 95% CI [0.40, 0.86], *p* < 0.01), and shorter duration of hospital stay (WMD = − 5.87 days, 95% CI [− 11.35, − 0.40], *p* < 0.05); however, the operation time was significantly longer in the laparoscopic group (WMD = 47.34 min, 95% CI [4.10, 90.58], *p* < 0.05). No other significant differences were observed.

**Conclusion:**

Laparoscopic ISR for low rectal cancer offers fewer complications and faster recovery, with similar operation quality and mid-term oncological results than the conventional approach. Although this technique is comparatively more complex than the conventional approach and requires practice, laparoscopic ISR shows great potential as a surgical option and deserves further clinical study.

## Background

Intersphincteric resection (ISR) was first introduced as an anus-preserving operation for very low rectal cancer approximately two decades ago [1]. Although it was initially utilized to treat inflammatory bowel disease [2], its performance in extirpating tumors and preserving anal function has been supported by much research [3–10]. Compared to classical abdominoperineal resection (APR), ISR with coloanal anastomosis obtained sufficient distal resection margins (DRM) and circumferential resection margins (CRM) and better protected anal function without necessitating a permanent colostomy [9–15].

Minimally invasive techniques and devices have rapidly progressed and now play increasingly important roles in surgery. In 1991, Dr. Jacobs first reported on laparoscopic rectal cancer surgery performance. The CLASSIC (comparing the Conventional versus Laparoscopic-Assisted Surgery in Colorectal Cancer) trial, the first clinical trial for this procedure, showed a significantly higher CRM-positive rate for laparoscopic surgery than open surgery (12 vs 6%) [16]. Although the following long-term outcomes in this trial and others have obtained similar or better survival data [16, 17], laparoscopic rectal surgery remains controversial.

Laparoscopic ISR combines laparoscopic total mesorectal excision (TME) and perineal resection. Thus, laparoscopic ISR is worth studying to determine whether patients can receive the benefits of common laparoscopic surgery, such as less trauma and postoperative pain, faster recovery, and shorter hospital stays. Many clinical studies have been undertaken on this new technique, among which the results and conclusions on different aspects of post-operational morbidity, oncological results, and functional recovery are inconsistent [18–28]. In this article, we comprehensively review the related controlled studies and perform a meta-analysis to provide evidence for evaluating the effects of laparoscopic ISR in low rectal cancer.

## Methods

### Search strategy

Studies published in English before March 12, 2017, were searched in the databases of PubMed, Embase, Ovid, and Cochrane using the main search terms “intersphincteric resection,” “ISR,” “laparoscopic surgery,” and “rectal cancer”. The search strategy differed per database by their different requirements. Additionally, relevant studies in the references of related articles were also screened.

### Inclusion and exclusion criteria

Studies with the following criteria were included:

1. The controlled study compared ISR using laparoscopy with the conventional open approach for low rectal cancer.
2. The article language was restricted to English.
3. Full text was available, and the compared outcomes contained at least one of the items mentioned below.
4. If the same research team participated in multiple studies, only the study with the most comprehensive data was included.

Studies were excluded for the following reasons:

1. Data on the main outcomes were unavailable.
2. Patients underwent ISR for reasons other than rectal cancer (e.g., rectal adenoma or proctitis).

### Data extraction and study quality assessment

Two researchers (Hanyu Chen and Bin Ma) evaluated the articles and extracted the data independently. Disagreements between the two researchers were presented to the third author and decided by consensus. The primary research outcomes of this meta-analysis were operation time, blood loss, CRM-positive rate, distal margin length, number of resected lymph nodes, postoperative overall morbidity, anastomotic leakage, and hospital stay. We also compared disease-free survival (DFS), overall survival (OS), and local recurrence as secondary outcomes to evaluate the mid-term effects of laparoscopic surgery.

### Statistical analysis

Statistical analysis was performed using Cochrane Collaboration software (RevMan v5.3; Nordic Cochrane Centre). All continuous variables were analyzed by the weighted mean difference (WMD). If mean values or standard deviation (SD) were not provided in the article, they were derived from the median values and ranges using the method described by Hozo et al. [29]. Dichotomous variables were evaluated by odds ratios (OR). Hazard ratios (HR) for DFS, OS, and local recurrence were calculated using software designed by Tierney et al. [30], and *p* < 0.05 was considered significant. Heterogeneity was obtained by the *χ* ^2^ test or the Cochran *Q* statistic, and the degree was measured by the *I* ^2^ value. If the *p* value of the *χ* ^2^ test is > 0.10 and *I* ^2^ < 50%, a fixed-effect model was used for non-significant heterogeneity; otherwise, the random-effect model was used in the meta-analysis for significant heterogeneity.

## Results

### Selected studies

**Fig. 1:**
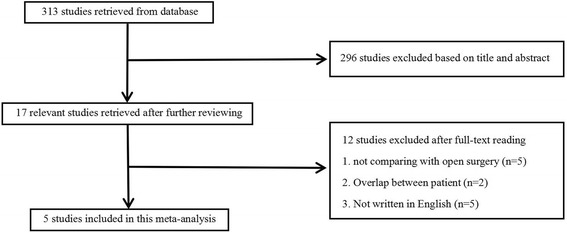
Flow chart showing the process for selecting the included studies

Three hundred thirteen studies were identified by the search strategy previously described (Fig. 1). Seventeen pertinent studies were found after reading the abstracts. Finally, five controlled clinical trials [18, 24–26, 28] were considered eligible after they were found to fit the inclusion criteria upon reading the full text. The studies were from Italy, Japan, China, and Korea. In total, 620 patients underwent ISR by laparoscopic surgery (*n* = 386) or open approach (*n* = 234) between all analyses (Table 1). All studies received Newcastle-Ottawa Scale scores ≥ 5 (Table 2).

**Table 1:** Basic characteristics of the included studies

Study	Year	Country	Type	Patients	Age mean ± SD/median (range)		BMI mean ± SD/median (range)		Pathological stage^a^				T stage^a^				N stage^a^				Mean tumor size mean ± SD/median (range)	
				Lap/open	Lap	Open	Lap	Open	Lap		Open		Lap		Open		Lap		Open		Lap	Open
									I/II III/IV		I/II III/IV		T0/1/2	3/4	T0/1/2	3/4	N0 1/2		N0 1/2			
Laurent [24]	2012	Italy	RCNT	110/65	64 (22–82)	64 (30–86)	24.4 (17–33)	25.3 (16–35)	76	34	48	17	16	94	9	56	49	61	33	32	NR	
Yamamoto [25]	2011	Japan	RCNT	22/22	55 (34–68)	58 (35–69)	21.8 (16.8–26.7)	22.5 (19.3–28.9)	18	4	19	3	NR				NR				2.2 (1.5–3.8)	2.8 (1.1–5.5)
Park [26]	2011	Korea	RCNT	130/80	60.9 ± 11.7	59.1 ± 11.4	23.4 ± 3.2	23.3 ± 2.6	76	54	44	36	53	77	46	34	NR				4.0 ± 1.9	4.3 ± 1.8
Chi [18]	2015	China	RCNT	89/48	56.8 ± 12.0	55.3 ± 14.9	22.2 ± 4.2	27.6 ± 6.5	36	53	16	32	46	43	26	22	63	26	26	22	3.4 ± 1.1	3.1 ± 1.4
Fujimoto [28]	2010	Japan	RCNT	35/19	61 (33–82)	58 (28–67)	NR	NR	19	14	14	5	22	13	8	11	18	17	10	9	2.7 (1.0–9.0)	3.5 (1.2–7.0)

^a^UICC classification

*SD* standard deviation, *BMI* body mass index, *RCNT* retrospective comparative non-randomized trial, *LAP* laparoscopic surgery, *Open* open surgery

**Table 2:** The risk of bias for the included studies (Newcastle-Ottawa scale)

Study	Selection				Comparability		Outcome			Total
	REC	SNEC	AE	DO	SC	AF	AO	FU	AFU	
Laurent [24]	1	0	1	1	0	0	1	1	1	6
Yamamoto [25]	1	1	1	1	1	1	1	0	1	8
Park [26]	1	1	1	1	0	0	1	1	1	7
Chi [18]	1	1	1	1	0	0	1	1	1	7
Fujimoto [28]	1	1	1	1	0	0	1	0	1	6

*REC* representativeness of the exposed cohort, *SNEC* selection of the non-exposed cohort, *AE* ascertainment of exposure, *DO* demonstration that outcome of interest was not present at start of study, *SC* study controls for age and sex, *AF* study controls for any additional factors (chemoradiotherapy, curative resection), *AO* assessment of outcome, *FU* follow-up long enough for outcomes to occur, *AFU* adequacy of follow-up of cohorts

### Operation time

**Fig. 2:**
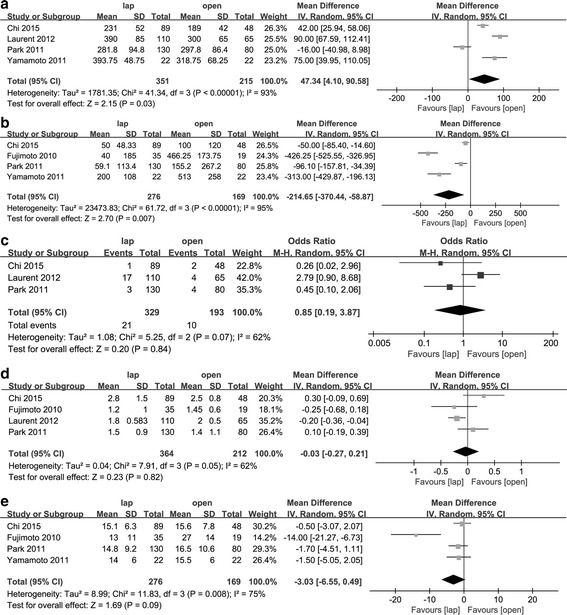
Forest plot based on short-term surgical outcomes. **a** Forest plot of the mean difference in operation time. **b** Forest plot of the mean difference in blood loss. **c** Forest plot of OR for a positive CRM. **d** Forest plot of the mean difference in DRM. **e** Forest plot of the mean difference in resected lymph node numbers

All five studies reported the operation time. Notably, the outcome from the study of Fujimoto et al. significantly deviated from the others. Upon comparing the procedures, we found that this group performed more lateral lymph node dissections in the open group, which is time-consuming. Thus, this study was not included in this outcome. Analyzing the other four studies revealed a significantly longer operation time for the laparoscopic surgery group (WMD = 47.34 min, 95% CI [4.10, 90.58], *p* < 0.05). A random-effect model was used due to the significant heterogeneity (*p* < 0.01, *I* ^2^ = 93%) (Fig. 2a).

### Blood loss

Four studies reported that significantly more blood was lost in the open group compared to the laparoscopic group (WMD = − 214.65 ml, 95% CI [− 370.44, − 196.13], *p* < 0.01). A random-effect model was used due to significant heterogeneity (*p* < 0.01, *I* ^2^ = 95%) (Fig. 2b).

### Positive CRM

A positive CRM occurs when the closest distance from the tumor to the resected mesorectal border is shorter than 1 mm. Although the study of Fujimoto et al. supplied the median and range of the CRM distance, precise numbers were not disclosed. Three studies provided sufficient CRM data. The meta-analysis results indicated no significant difference between the two groups (OR = 0.85, 95% CI [0.19, 3.87], *p* = 0.84). A random-effect model was used due to heterogeneity (*p* = 0.07, *I* ^2^ = 62%) (Fig. 2c).

### Distal resected margin

All studies except for that of Yamamoto et al. compared the length of the DRM from the tumor. Pooled results suggested no significant difference between the two groups. (WMD = − 0.03 cm, 95% CI [− 0.27, 0.21], *p* = 0.82). A random-effect model was used due to significant heterogeneity (*p* = 0.05, *I* ^2^ = 62%) (Fig. 2d).

### Lymph nodes resected

All studies except for that of Laurent et al. provided the number of harvested lymph nodes. After pooling the results, we found no significant difference between the two groups (WMD = − 3.03, 95% CI [− 6.55, 0.49], *p* = 0.09). A random-effect model was used due to significant heterogeneity (*p* < 0.01, *I* ^2^ = 75%) (Fig. 2e).

### Overall morbidity

**Fig. 3:**
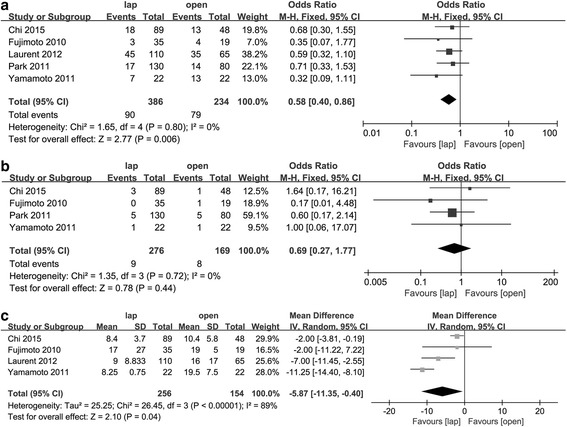
Forest plot based on short-term recovery outcomes. **a** Forest plot of OR for overall morbidity. **b** Forest plot of OR for anastomotic leakage. **c** Forest plot of the mean difference in hospital stay length

All studies researched the post-operational complication incidence. The overall morbidity between each study showed no significant differences; however, after the meta-analysis, the results showed a significantly decreased occurrence rate in the laparoscopic group (OR = 0.58, 95% CI [0.40, 0.86], *p* < 0.01). A fixed-effect model was used due to a lack of remarkable heterogeneity (*p* = 0.80, *I* ^2^ = 0%) (Fig. 3a).

### Anastomotic leakage

As the most important surgery-related complication, we extracted data on anastomotic leakage separately from overall morbidity. Four studies supplied this specific data, and no significant difference was found between the two groups for anastomotic leakage incidence (OR = 0.69, 95% CI [0.27, 1.77], *p* = 0.44). No significant heterogeneity was noted; thus, the fixed-effect model was used (*p* = 0.72, *I* ^2^ = 0%) (Fig. 3b).

### Hospital stay

Four studies reported the hospital stay duration. The laparoscopic surgery resulted in significantly shorter hospital stays than the open surgery (WMD = − 5.87 days, 95% CI [− 11.35, − 0.40], *p* < 0.05). A random-effect model was used due to significant heterogeneity (*p* < 0.01, *I* ^2^ = 89%) (Fig. 3c).

### DFS, OS, and local recurrence

**Fig. 4:**
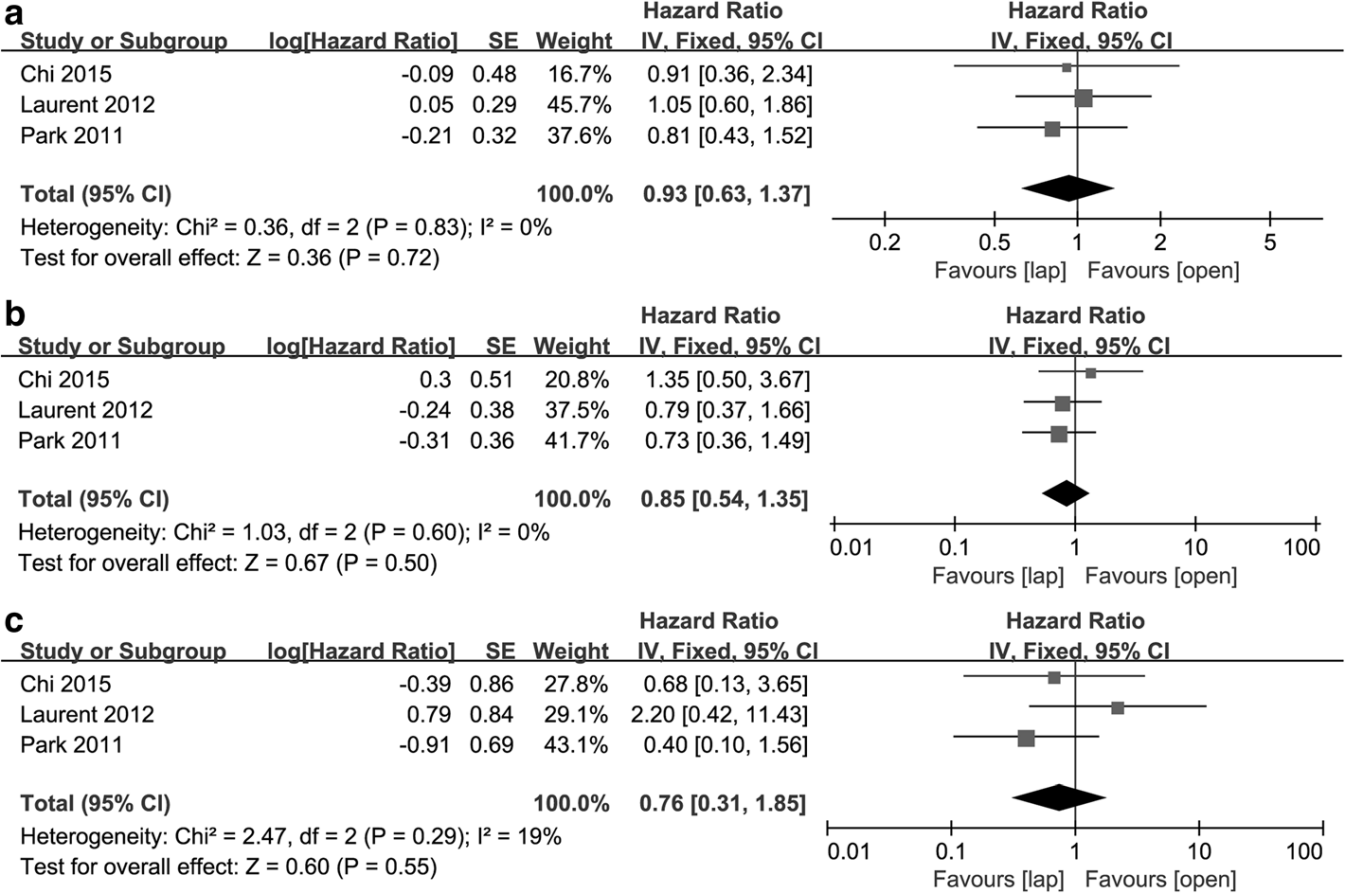
Forest plot based on mid-term outcomes. **a** Forest plot of HR for DFS. **b** Forest plot of HR for OS. **c** Forest plot of HR for local recurrence

Three studies provided data regarding mid-term efficacy between the two groups. As the three main indexes of survival, DFS, OS, and local recurrence were calculated. The results suggested no significant difference between the two group in DFS (HR = 0.93, 95% CI [0.63, 1.37], *p* = 0.72), OS (HR = 0.85, 95% CI [0.54, 1.35], *p* = 0.50), or local recurrence (HR = 0.76, 95% CI [0.31, 1.85], *p* = 0.55). No remarkable heterogeneity was seen in these items (DFS: *p* = 0.83, *I* ^2^ = 0%, OS: *p* = 0.60, *I* ^2^ = 0%, local recurrence: *p* = 0.29, *I* ^2^ = 19%) (Fig. 4).

### Post-operational anal function and diverting stoma rate

Comparative data for these two outcomes could only be acquired from one or two studies which was insufficient for a convincing meta-analysis; nevertheless, we reviewed these data in the “Discussion” section.

## Discussion

Laparoscopic surgery has gradually been accepted as a minimally invasive technique because of its decreased blood loss, reduced incision-related complications, and faster recovery. The feasibility and safety of this technique in treating rectal cancer has been demonstrated [17, 31]. Particularly in the low rectum, surgeons have found that laparoscopic surgery provides better visibility when operating in the lower pelvic cavity such as when dissecting the mesorectum plane, dividing lateral ligaments, mobilizing the pelvic floor, or dissecting the intersphincteric groove [32, 33]. This procedure also allows the pelvic autonomic nerves related to defecation, urination, and sexual function to be easily seen and well-preserved under the camera. Whether patients receive increased benefits from laparoscopic surgery compared with conventional open surgery in ISR remains controversial. Thus, we conducted a meta-analysis to ascertain whether the advantages of laparoscopic surgery provide better short- and intermediate-term outcomes and whether the shortcomings of this technique may be harmful.

The short-term results revealed that operation times were longer for laparoscopic surgery. Procedural complexity is unavoidable, leading many to resort to open surgery or endure longer operation times before the surgeon gains sufficient practice [34–36]. However, these technical difficulties can be surmounted as the surgeons’ experience increases. Kuo et al. [19] compared the short-term outcomes of patients who received the laparoscopic ISR procedure during the initial 18 months in which they began performing the procedure to those of patients who underwent this surgery after the initial 18 months. They found that operation times were significantly shorter, and significantly more lymph nodes were retrieved, as the surgeon gained more experience. Future studies are needed to reveal the learning curve pattern and provide surgeons with an expected timeframe for becoming more experienced.

Significantly less blood was lost in every study that used the laparoscopic approach without conflicts, which demonstrates the impressive advantages of this minimally invasive technique. The CRM-positive rate, DRM length, and number of resected lymph nodes were similar, indicating a similar extent of radical resection. Postoperative overall morbidity, including incision, anastomotic and systemic complications, was lower with laparoscopic surgery. Among overall morbidity, anastomotic leakage was closely related to operation quality. No differences were seen between the two surgical approaches. Furthermore, the hospital stay length was shorter in the laparoscopic group. The shorter convalescence and decreased complications reflect that this minimally invasive technique is also advantageous for ISR.

The oncological survival and local recurrence outcomes are undoubtedly the most important evaluation indicators for tumor operations. The mid-term outcomes of DFS, OS, and local recurrence in the two groups showed no apparent differences. We must note that follow-up data could only be compiled for 3 years from the article date to compare mid-term outcomes. Most recent studies comparing laparoscopic and open surgery for colorectal cancer use 3-year oncological results as primary outcomes [17, 37]. However, no trials have found patients who were DFS-negative at the 3-year follow-up become positive at the 5-year follow-up; thus, these similar mid-term outcomes largely confirm the beneficial effects of laparoscopic surgery.

Compared with conventional APR, ISR is notably advantageous for avoiding permanent colostomy and protecting anal function. However, only two studies [18, 26] compared the post-operational anal function between laparoscopic surgery and the open approach by the Wexner score, and the follow-up and evaluation details were not provided. Although similar results were obtained in these two studies, and no significant differences were found, meta-analysis results are unconvincing.

Diverting stoma as a post-anastomosis option in the low rectal surgery remains controversial in ISR. Both in laparoscopic ISR [18, 27] and open surgery [7, 8, 10], the diverting stoma was routinely practiced in many surgical units, to divert the feces and keep the anastomosis from being contaminated to avoid leakage and allow time for sphincter-strengthening exercises to improve anal function. However, Park et al. [26] practiced only a 10.6% rate of diverting stoma in the laparoscopic ISR group and obtained an overall leakage rate of 3.8%, which was comparatively lower than the routine ileostomy results following open ISR. These results may have been due to the good anal vascularity and the hand-sewn coloanal anastomosis method providing good edge-to-edge apposition without luminal diameter deviation. More specific indications for using the diverting stoma should be further investigated.

Several limitations remain in this work. First, only non-randomized studies were available in this field, which may reduce each result’s reliability. Second, the limited number of applicable studies may influence the statistical power. Third, the experience and skill of each surgeon likely differed between studies, which could provide unavoidable bias.

## Conclusions

In conclusion, compared to the conventional open approach, laparoscopic ISR for low rectal cancer has the advantages of fewer complications and faster recovery along with a similar operation quality and mid-term oncological results. Although this technique is comparatively more complex than the conventional open approach and requires extensive practice, laparoscopic ISR has great potential as a surgical option and warrants further clinical research.

## Abbreviations

APR: Abdominoperineal resection
BMI: Body mass index
CRM: Circumferential resection margin
DFS: Disease-free survival
DRM: Distal resection margin
HR: Hazard ratios
ISR: Intersphincteric resection
OR: Odds ratios
OS: Overall survival
RCNT: Retrospective comparative non-randomized trial
SD: Standard deviation
TME: Total mesorectal excision
WMD: Weighted mean difference

## References

[1] Braun J, Treutner KH, Winkeltau G, Heidenreich U, Lerch MM, Schumpelick V (1992). Results of intersphincteric resection of the rectum with direct coloanal anastomosis for rectal carcinoma. Am J Surg.

[2] Lyttle JA, Parks AG (1977). Intersphincteric excision of the rectum. Br J Surg.

[3] Teramoto T, Watanabe M, Kitajima M (1997). Per anum intersphincteric rectal dissection with direct coloanal anastomosis for lower rectal cancer: the ultimate sphincter-preserving operation. Dis Colon Rectum.

[4] Rullier E, Zerbib F, Laurent C, Bonnel C, Caudry M, Saric J, Parneix M (1999). Intersphincteric resection with excision of internal anal sphincter for conservative treatment of very low rectal cancer. Dis Colon Rectum.

[5] Köhler A, Athanasiadis S, Ommer A, Psarakis E (2000). Long-term results of low anterior resection with intersphincteric anastomosis in carcinoma of the lower one-third of the rectum: analysis of 31 patients. Dis Colon Rectum.

[6] Saito N, Ono M, Sugito M, Ito M, Morihiro M, Kosugi C, Sato K, Kotaka M, Nomura S, Arai M, Kobatake T (2004). Early results of intersphincteric resection for patients with very low rectal cancer: an active approach to avoid a permanent colostomy. Dis Colon Rectum.

[7] Schiessel R, Novi G, Holzer B, Rosen HR, Renner K, Hölbling N, Feil W, Urban M (2005). Technique and long-term results of intersphincteric resection for low rectal cancer. Dis Colon Rectum.

[8] Chamlou R, Parc Y, Simon T, Bennis M, Dehni N, Parc R, Tiret E (2007). Long-term results of intersphincteric resection for low rectal cancer. Ann Surg.

[9] Saito N, Sugito M, Ito M, Kobayashi A, Nishizawa Y, Yoneyama Y, Nishizawa Y, Minagawa N (2009). Oncologic outcome of intersphincteric resection for very low rectal cancer. World J Surg.

[10] Yamada K, Ogata S, Saiki Y, Fukunaga M, Tsuji Y, Takano M (2009). Long-term results of intersphincteric resection for low rectal cancer. Dis Colon Rectum.

[11] Barisic G, Markovic V, Popovic M, Dimitrijevic I, Gavrilovic P, Krivokapic Z (2011). Function after intersphincteric resection for low rectal cancer and its influence on quality of life. Color Dis.

[12] Kuo LJ, Hung CS, Wu CH, Wang W, Tam KW, Liang HH, Chang YJ, Wei PL (2011). Oncological and functional outcomes of intersphincteric resection for low rectal cancer. J Surg Res.

[13] Zhang YJ, Yin L, Huang L, Zhang HB, Han Y, Lin MB (2013). Long-term results of intersphincteric resection for low rectal cancer. J Investig Surg.

[14] Giglio MC, Persico M, Quarto G, Benassai G, Luglio G, Tarquini R, Celentano V, Sollazzo V, Bucci L (2013). Intersphinteric resection for rectal cancer: role in fecal continence and quality of life. Ann Ital Chir.

[15] Konanz J, Herrle F, Weiss C, Post S, Kienle P (2013). Quality of life of patients after low anterior, intersphincteric, and abdominoperineal resection for rectal cancer—a matched-pair analysis. Int J Color Dis.

[16] Green BL, Marshall HC, Collinson F, Quirke P, Guillou P, Jayne DG, Brown JM (2013). Long-term follow-up of the Medical Research Council CLASICC trial of conventional versus laparoscopically assisted resection in colorectal cancer. Br J Surg.

[17] van der Pas MH, Haglind E, Cuesta MA, Fürst A, Lacy AM, Hop WC, Bonjer HJ (2013). Colorectal cancer Laparoscopic or Open Resection II (COLOR II) study group. Laparoscopic versus open surgery for rectal cancer (COLOR II): short-term outcomes of a randomised, phase 3 trial. Lancet Oncol..

[18] Chi P, Huang SH, Lin HM, Lu XR, Huang Y, Jiang WZ, Xu ZB, Chen ZF, Sun YW, Ye DX (2015). Laparoscopic transabdominal approach partial intersphincteric resection for low rectal cancer: surgical feasibility and intermediate-term outcome. Ann Surg Oncol.

[19] Kuo LJ, Hung CS, Wang W, Tam KW, Lee HC, Liang HH, Chang YJ, Huang MT, Wei PL (2013). Intersphincteric resection for very low rectal cancer: clinical outcomes of open versus laparoscopic approach and multidimensional analysis of the learning curve for laparoscopic surgery. J Surg Res.

[20] Mukai M, Sekido Y, Fukumitsu H, Izumi H, Hoshikawa T, Tajima T, Tobita K, Sadahiro S, Yasuda S, Ogoshi K (2011). Anal function-preserving subtotal intersphincteric resection/partial external sphincteric resection with hybrid 2-port hand-assisted laparoscopic surgery (Mukai’s operation) for very low stage I rectal cancer: a case report. Oncol Lett.

[21] Pai VD, De Souza A, Patil P, Engineer R, Arya S, Saklani A (2015). Intersphincteric resection and hand-sewn coloanal anastomosis for low rectal cancer: short-term outcomes in the Indian setting. Indian J Gastroenterol.

[22] Scala D, Niglio A, Pace U, Ruffolo F, Rega D, Delrio P (2016). Laparoscopic intersphincteric resection: indications and results. Updat Surg.

[23] Funahashi K, Shiokawa H, Teramoto T, Koike J, Kaneko H (2011). Clinical outcome of laparoscopic intersphincteric resection combined with transanal rectal dissection for T3 low rectal cancer in patients with a narrow pelvis. Int J Surg Oncol.

[24] Laurent C, Paumet T, Leblanc F, Denost Q, Rullier E (2012). Intersphincteric resection for low rectal cancer: laparoscopic vs open surgery approach. Color Dis.

[25] Yamamoto S, Fujita S, Akasu T, Inada R, Takawa M, Moriya Y (2011). Short-term outcomes of laparoscopic intersphincteric resection for lower rectal cancer and comparison with open approach. Dig Surg.

[26] Park JS, Choi GS, Jun SH, Hasegawa S, Sakai Y (2011). Laparoscopic versus open intersphincteric resection and coloanal anastomosis for low rectal cancer: intermediate-term oncologic outcomes. Ann Surg.

[27] Lim SW, Huh JW, Kim YJ, Kim HR (2011). Laparoscopic intersphincteric resection for low rectal cancer. World J Surg.

[28] Fujimoto Y, Akiyoshi T, Kuroyanagi H, Konishi T, Ueno M, Oya M, Yamaguchi T (2010). Safety and feasibility of laparoscopic intersphincteric resection for very low rectal cancer. J Gastrointest Surg.

[29] Hozo SP, Djulbegovic B, Hozo I (2005). Estimating the mean and variance from the median, range, and the size of a sample. BMC Med Res Methodol.

[30] Tierney JF, Stewart LA, Ghersi D, Burdett S, Sydes MR (2007). Practical methods for incorporating summary time-to-event data into meta-analysis. Trials.

[31] Kang SB, Park JW, Jeong SY, Nam BH, Choi HS, Kim DW, Lim SB, Lee TG, Kim DY, Kim JS, Chang HJ, Lee HS, Kim SY, Jung KH, Hong YS, Kim JH, Sohn DK, Kim DH, Oh JH (2010). Open versus laparoscopic surgery for mid or low rectal cancer after neoadjuvant chemoradiotherapy (COREAN trial): short-term outcomes of an open-label randomised controlled trial. Lancet Oncol.

[32] Hamada M, Matsumura T, Matsumoto T, Teraishi F, Ozaki K, Nakamura T, Fukui Y, Nishioka Y, Taniki T, Horimi T (2011). Video. Advantages of the laparoscopic approach for intersphincteric resection. Surg Endosc.

[33] Huh JW (2014). Minimally invasive techniques for an intersphincteric resection and lateral pelvic lymph node dissection in rectal cancer. Ann Coloproctol.

[34] Rullier E, Sa Cunha A, Couderc P, Rullier A, Gontier R, Saric J (2003). Laparoscopic intersphincteric resection with coloplasty and coloanal anastomosis for mid and low rectal cancer. Br J Surg.

[35] Shiomi A, Kinugasa Y, Yamaguchi T, Tsukamoto S, Tomioka H, Kagawa H (2013). Feasibility of laparoscopic intersphincteric resection for patients with cT1-T2 low rectal cancer. Dig Surg.

[36] Fujii S, Yamamoto S, Ito M, Yamaguchi S, Sakamoto K, Kinugasa Y, Kokuba Y, Okuda J, Yoshimura K, Watanabe M (2012). Short-term outcomes of laparoscopic intersphincteric resection from a phase II trial to evaluate laparoscopic surgery for stage 0/I rectal cancer: Japan Society of Laparoscopic Colorectal Surgery Lap RC. Surg Endosc.

[37] Jayne DG, Guillou PJ, Thorpe H, Quirke P, Copeland J, Smith AM, Heath RM, Brown JM, UK MRC CLASICC Trial Group (2007). Randomized trial of laparoscopic-assisted resection of colorectal carcinoma: 3-year results of the UK MRC CLASICC trial group. J Clin Oncol.

